# A Transmission-Based Dielectric Property Probe for Clinical Applications

**DOI:** 10.3390/s18103484

**Published:** 2018-10-16

**Authors:** Paul Meaney, Tomas Rydholm, Helena Brisby

**Affiliations:** 1 Electrical Engineering Department, Chalmers University of Technology, 41296 Gothenburg, Sweden; tomas.rydholm@chalmers.se; 2 Thayer School of Engineering, Dartmouth College, Hanover, NH 03755, USA; 3 Sahlgrenska University Hospital, 41345 Gothenburg, Sweden; helena.brisby@vgregion.se

**Keywords:** open-ended coaxial cable, penetration depth, transmission-based dielectric probe, vertebra bone

## Abstract

We have developed a transmission-based, open-ended coaxial dielectric probe that can be used in clinical situations and overcomes many of the limitations related to the typical reflection-based dielectric probes. The approach utilizes the low profile, open-ended coaxial cables enabling clinicians to still probe relatively compact spaces. The sensing depth can be extended to as large as 1.5 to 2 cm compared with the more typical range of 0.3 mm for conventional probes and is dramatically less affected by measurement technique variability including poor sample contact and cable bending. As a precursor to an actual clinical implementation, we study the technique in a range of homogeneous liquids with substantially varying dielectric properties. The initial results demonstrate good agreement between the transmission-based probe and commercial, reflection-based probes and pave the way for more substantial clinical implementation.

## 1. Introduction

The reflection based, open-ended coaxial dielectric probe has found wide use in industry and medical applications over nearly four decades and essentially provides ground truth for many studies [1,2,3,4,5,6,7]. Numerous calibration models have been developed over time which provide accurate results over a large range of frequencies [8,9,10,11,12,13]. Their use is particularly beneficial when there is only access to one side of an object. In the case of tissue measurements, their use can dramatically reduce the tissue preparation time which can be critical depending on study needs—in particular because of the substantial change in properties immediately after excision [14,15,16]. The most widely used commercial system is that developed by Keysight Technologies (Santa Clara, CA, USA) [17] but more recent products are now marketed by Delfin Technologies (Kuopio, Finland) [18,19] and SPEAG (Zurich, Switzerland) [20]. While the Keysight technology has proved useful in various settings; however, recent studies have shown that the penetration depth is directly related to the probe diameter which has proven detrimental in various tissue characterization studies since their effective probe diameter is only on the order of 2 mm [18,21,22,23]. Typical penetration depths are on the order of 1/6th that of the probe diameter [23]. The SPEAG technology has solved some concerns regarding cable bending issues by attaching the probe directly to the microwave electronics device and by introducing larger diameter probes for deeper penetration [20]. The former now allows for a more handheld operation which provides further benefits. This larger probe is very useful in its intended application of placing the probe on the skin surface and determining the level of oedema down to diagnostically useful depths [19]. However, there are critical in vivo applications where having a small probe is useful while also requiring the ability to assess properties over a space of roughly 1cm across or larger—as will be discussed below.

The penetration depth is a particular concern for this technology. In the case of measuring heterogeneous tissue, the Keysight probes only sense roughly the top 280 μm into the surface [21,22]. It is easily conceivable that the top tissue layer might not be representative of the entire excised tissue. This is also precisely where the steepest temperature gradients are for newly excised tissue. Additionally, very often newly excised tissue is covered with a thin layer of blood that can either be fresh or dried depending on the technician tissue preparation processes. With the sensing depth being so shallow, the blood properties could easily confound the recovered composite properties. These factors and others can easily impact the desired measurements.

Reflection-based, horn antenna approaches have also been employed typically for characterization of thinly layered materials [24]. These have typically utilized thin layers of material positioned at a known distance in front of the horn and a reflection plate at varying distances behind the sample. Most notably, these have found use in characterizing polymer layers. Finally, reflection-based interferometry and microwave microscopy techniques have recently been introduced by Bakli and Haddadi [25,26] which were used to very accurately assess the changes in saline properties as a function of sodium chloride concentration at two single frequencies and general determination of the dielectric constants and loss tangents of liquids at 2.45 GHz.

Alternatively, a range of transmission techniques have been used to measure the dielectric properties of materials. One of the more common approaches is to insert the sample into either a waveguide or coaxial chamber and assess the properties based on perturbations to the S-parameters. Harris et al. [27] performed a useful comparison of the waveguide and coaxial approaches which concluded that the waveguide techniques were easier to manipulate because of the required size and shape of the sample but that the coaxial technique had bandwidth advantages because it was not constricted by operating cut-off frequencies. Numerous studies by Nicolson and Ross [28], Weir [29] and Larsson et al. [30] have thoroughly analysed the problem in terms of deriving the complex permittivity and permeability values based on the corresponding measured S-parameters. Correspondingly, researchers have also configured free space set ups for similar electrical property measurements. These typically involve two directive horn antennas oriented towards each other and a known thickness sample placed between them [31,32,33]. The changes in the measured transmission phase and amplitude are used to infer what the properties of the sample must have been. One interesting variant of this is the technique developed by Garret and Fear [34], where they use two opposing aperture antennas directed at each other and slightly compress the breast of a patient between them. Utilizing ultra-wideband transmission, they are able to recover rough estimates of the overall dielectric property distribution which includes both the adipose and fibroglandular tissues. A more recent version of this approach has been described in Garrett et al. [35] utilizing both transmission and reflection S-parameter measurements to recover in vivo properties of the compressed breast. These values are subsequently used as initial estimates for inverse scattering imaging of the breast. While useful for certain structures, it is impractical in many settings. In general, these approaches are not suitable for in vivo surgical settings.

Transmission measurement techniques have also been a mainstay of tomographic and inverse scattering imaging approaches. The technique presented by Jacobi and Larsen [36] is noteworthy in that they recovered useful images; however, they introduced chirp measurement strategies which allowed them to directly decrease the impact of multipath signals.

We have developed a technique that utilizes some aspects of the transmission concepts but exploits the fact that the open-ended coaxial cables are so small that our measurements are effectively in the far field which has important ramifications for certain in vivo and ex vivo settings. This transmission-based dielectric measurement scheme utilizing two opposing open-ended coaxial cables. Similarly, to the two, large antenna approach, this method measures the amplitudes and phases for signals traversing the full intervening volume to provide a more representative measure of the properties throughout the volume. However, because the receiving antenna is in the far field, simplified equations can be used to extract the associated dielectric properties without a complete S-parameter analysis. The primary challenge is that the signal reception can be weak depending on the probe separation, frequency and the lossiness of the sample. Generally, only fringing fields escape from the ends of the open-ended coaxial cables. The probes are essentially low efficiency antennas. However, beyond the previous benefit just mentioned, there are other critical advantages. First it is significantly less sensitive to probe contact with the sample. Additionally, owing to the fact that the reflection-based probe effectively operates at one of the two more unstable points on the Smith chart (i.e., an open circuit), the transmission based approach is quite stable even with respect to cable bending.

Two important drawbacks include the fact that a sample needs to be contacted on opposite sides by the probes and that the thickness cannot be that great. In this case we are generally considering thicknesses on the order of 1–2 cm. While these can be important limitations, we will discuss a specific application where this approach can be a real advantage, even for in vivo settings. Along with these issues is the need for a high dynamic range measurement system. The Rohde and Schwarz ZNBT8 system can readily measure down to -140dBm from a transmitted 1mW signal to easily meet the needs of this approach. Additionally, more compact concepts utilizing evolving software defined radios (SDR’s) are being developed to meet this type of need in a more compact and economical way [37].

One important scenario where we plan to apply this new concept is in the area of bone density measurements of vertebrae during spinal fusion surgeries. Back pain and injuries are significant health problems in North America and northern European countries [38]. In these cases, the spinal column needs to be held immobile for a lengthy period of time until adjoining vertebrae become substantially fused. For these situations, instrumentation is installed over a length of multiple vertebrae to hold the column in place. A very common type of instrumentation involves posterior pedicle screws connected by rods in several continuous vertebrae to supply sufficient support. It is critical that the vertebrae are strong enough to withstand the forces exerted through the screws [39]. While systemic bone density measures such as dual-energy X-ray absorptiometry (DXA) provide an indicator of strength, the overall surgical success rate is decreased in bone with low density [40,41]. Loosening instrumentation failure can lead to significant patient pain and inconvenience, trauma and high costs of repeated surgeries [39,42,43,44]. While exact prevalence of and health care costs associated with osteoporotic-caused surgery failures is not known, it is certainly acknowledged as a significant medical problem [39,44]. Direct measures of the bone tissue during surgery could assist in the decision making and thereby minimize the number of repeated surgeries, reducing health care costs while improving overall patient quality of life. In cases where the dielectric probe determines that the vertebrae are weak, alternative surgical strategies can be used to improve the probability of success. While there are methods to improve the strength of the instrumentation such as by either incorporating more vertebrae or utilizing specialized screws and/or anatomical cements with the screws, each presents their own risks. Adding more bones simply increases the invasiveness of the surgical procedure. In addition, there are risks that small pieces of the dried cement could get into the blood stream leading to embolisms.

While DXA scans are generally regarded as a gold standard for bone density determination, there are numerous factors which can confound these results and produce misleading measurement values and lead to inappropriate treatments [40,41]. A typical DXA reading of the spine is often taken as an average of values from three to four continuous lumbar vertebrae. This immediately implies a level of local variation and, especially if the measurement is performed in the anterior-posterior view, osteoarthritis of the facet joints and local osteophytes may highly influence the values. In addition, there are reports of significant variation between technicians who perform the exams. Equally importantly, because DXA readings are based on single projection X-rays, a variety of factors can influence the readings. These can include artefacts from different calcifications in some of the overlying tissue such as the aorta and pancreas and, for the thoracic spine, the rib cage makes measurement of the vertebra almost impossible.

As mentioned above, the most common approach for constructing stabilizing instrumentation of part of the spine is to fixate the vertebra by inserting screws through canals in the pedicles and continuing on into the central mass of the vertebrae [39,42]. In cases where the bones are insufficiently strong, there are alternatives. While bio-compatible cements can be used to strengthen the bone:screw interface as mentioned above, other approaches include using specially designed screws or involving more vertebrae to reduce the physical load on each screw [39,42]. This obviously requires a more invasive surgery with concomitant health risks.

A more definitive measure of vertebrae suitability on a bone by bone basis directly during the surgical session would be ideal for creating an individually designed surgical plan and performance. The new transmission probe concept may be ideal for this situation. Historically, dielectric probe measurements of bone have been challenging because of the different issues discussed above. These are further complicated by the fact that the measurements would need to be performed in vivo. This setting could prove particularly intriguing for the new probes because the clinician has access to the vertebrae from two sides through the opposing pedicle canals. While the orientation of a pair of standard open-ended coaxial cables would not be aligned perfectly, in a more refined implementation, simple, custom bends in the coax can be added to provide the desired configuration.

The remainder of this paper describes the theoretical underpinnings of the approach followed by phantom experiments to demonstrate overall feasibility. Section 2 discusses the theory and measurement configurations and Section 3 presents representative results. While this study discusses the theory with respect to the slopes of the signal amplitudes and phases requiring multiple measurements, a final product will ultimately only have access to a single measurement. Strategies for accommodating this challenge are left for further development.

## 2. Methods

### 2.1. Solving for Dielectric Properties from Amplitude and Phase Measurements

For this implementation, receiving a signal radiated from the open end of a 2.2 mm diameter coaxial cable and received from a similar cable, the threshold for the far field can be approximated as $2D^{2}$/λ, where D is the diameter of the aperture and *λ* is the wavelength. Using a diameter of 1.68 mm (the diameter of the coaxial cable insulator) and a maximum frequency of 8 GHz, the largest far field threshold would be 1.35 mm in the worst case when the medium was water. Given that the intended range is approximately between 10–20 mm, the far field approximation is valid. This substantially simplifies the computations compared with other transmission mode dielectric measurement techniques which required full S-parameter analysis. In general, the far field signals measured at one antenna due to a signal propagating from another are proportional to $1/r^{2}$ and $e^{-jkr}$, where *r* is the separation distance and *k* is the complex wavenumber. This relationship can be written as:

(1) $$\;E_{received}=C*\frac{e^{-jkr}}{r^{2}}=C*\frac{e^{-j\beta r-\alpha r}}{r^{2}}=\left[c_{1}\frac{e^{-\alpha r}}{r^{2}}\right]*\left[c_{2}e^{-j\beta r}\right]\;$$

where *C*, $c_{1}$ and $c_{2}$ are constants, *β* is the phase constant and *α* is the attenuation coefficient. The constants are included to simplify the equations and for completeness sake but are not included in the latter steps of the derivation. In this representation, the equation has been separated into the real and imaginary parts, respectively.

From the first of the last two terms, the amplitude (dB) can be written as:

(2) $$\begin{array}{cc} Magnitude & =20*\;log_{10}\left(c_{1}\right)-8.68589\alpha r-20*log_{10}\left(r^{2}\right)\\ & =c_{1a}-8.68589\alpha r-20*log_{10}\left(r^{2}\right) \end{array}$$

By adding a factor of $20*log_{10}\left(r^{2}\right)$ to this equation which is possible since the distance *r* is known, the resulting equation is:

(3) $$\;Magnitude=c_{1a}-8.68589\alpha \;$$

where $c_{1a}$ is a constant and the slope is just −8.68589*α*. Therefore, *α* can be computed directly from the measurement data as a function of the magnitude slope once the $20*log_{10}\left(r^{2}\right)$ term has been added. This is performed by measuring the data at multiple spacings, plotting the magnitude as a function of separation distance, adding the $20*log_{10}\left(r^{2}\right)$ factor and using a least squares parameter estimation technique to fit the data to a straight line. Spacings less than 1.5 mm were not included in the fitting process to minimize corruption from data points not within the far field. In addition, spacings greater than 20 mm were not used because of corruption from noise. The resulting slope, *α*, is used in these calculations. The constant $c_{1a}$ is not used but was included in the equation for completeness purposes.

Similarly, by taking the natural logarithm of the last term in Equation (1), we get:

(4) $$Phase=c_{2a}-\;\beta r\;$$

where $c_{2a}$ is a constant and *β* is the phase constant in radians/m. This is performed by measuring the data at multiple spacings, plotting the phase as a function of separation distance and using a least squares parameter estimation technique to fit the data to a straight line. The resulting slope, *β*, is used in these calculations. The constant $c_{2a}$ is not used but was included in the equation for completeness purposes.

Once *α* and *β* have been determined, the dielectric properties can be solved for directly:

(5) $$\;\beta -j\alpha =\sqrt{k^{2}}=\sqrt{\left(\omega^{2}\mu_{o}\varepsilon_{o}\right)\left(\varepsilon^{\prime }-j\varepsilon^{\prime \prime }\right)}\;$$

where *ω* is the frequency in radians, *μ*_o_ is the free space magnetic permeability, *ε_o_* is the free space electrical permittivity, *ε′* is the real permittivity, *ε″* is the imaginary permittivity and *j* is $\sqrt{-1}$, respectively. Equation (5) can be expanded to isolate both *ε′* and *ε″*, respectively:

(6) $$\varepsilon^{\prime }=\frac{\beta^{2}\;-\alpha^{2}}{\omega^{2}\mu_{o}\varepsilon_{o}}\;\mathrm{and}\;\varepsilon^{\prime \prime }=\frac{2\beta \alpha }{\omega^{2}\mu_{o}\varepsilon_{o}}\;$$

### 2.2. Measurement Configuration

**Figure 1:**
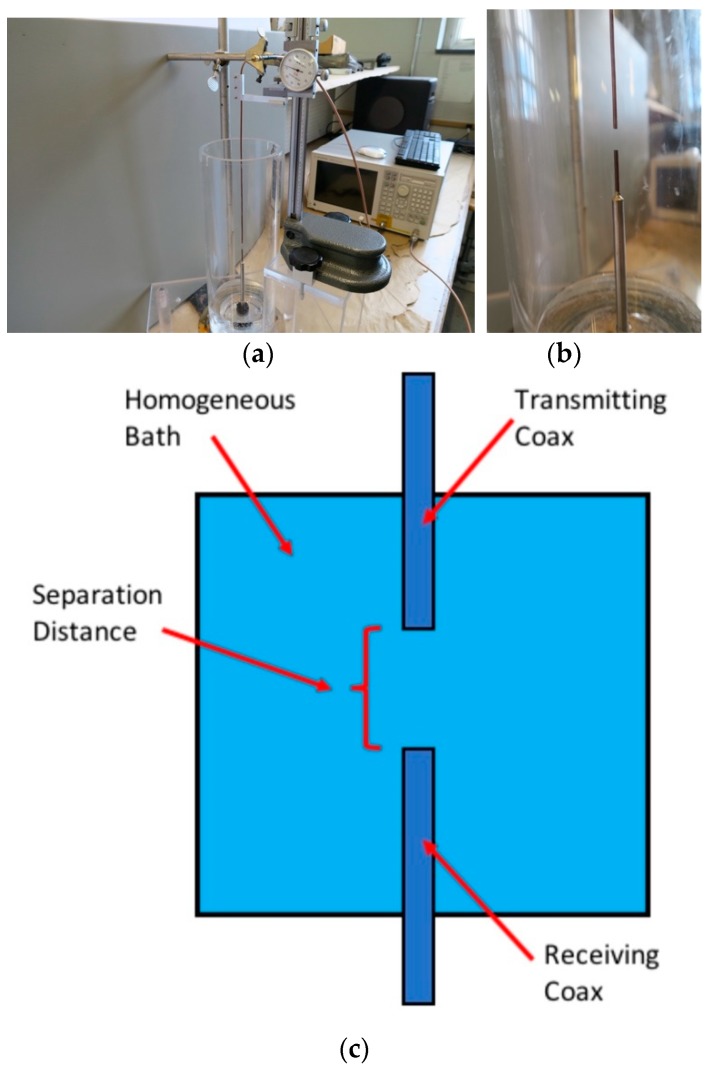
Photographs (**a**,**b**) and a schematic diagram (**c**) of the experimental set-up where the separation distances are accurately positioned through controlled motion of the calliper.

Figure 1a,b shows a photograph and schematic diagram of the transmission probe measurement experimental set-up. One semi-rigid coaxial cable protrudes vertically through the centre of the base of the 15.2 cm diameter tank. A second probe is attached to a calliper which is positioned at the top of the tank. Flexible coaxial cables were attached to both semi-rigid coaxes and fed into the Rohde and Schwarz vector network analyser (model ZNBT8—Munich, Germany). The system is calibrated using the internal device 2-port calibration process with the reference plane set at the interfaces of the two flexible coaxial cables attached to the two semi-rigid coaxial cables comprising the probes. S_21_ amplitude and phase data was acquired at 201 frequencies in increments from 9 KHz to 8.5 GHz with the IF bandwidth set to 1 KHz, along with the averaging set to a factor of 1 to maximize the dynamic range. The time for each acquisition was 0.2 s. Data was initially acquired for when the two open-ended coaxial cables were concentrically touching each other and then subsequently at 0.5 mm intervals up to a maximum separation of 4 cm. Data was acquired for three mixtures of glycerin and water because of its ability to produce a wide range of dielectric properties depending on the mixture ratios.

### 2.3. Error Considerations

For the clinical implementation, we expect three primary sources or error: (a) possible air gaps and poor coaxial cable/target contact, (b) errors in the distance measurements which will impact the slope calculations and (c) accuracy and consistency for the calibration measurements. For the former, these would be difficult to assess in an experimental configuration because the technique depends on good contact with the tissue (or liquid) along the outside of the coax to attenuate unwanted multipath signals. In such a situation, it would be difficult to accurately quantify the amount of dislocation between the probe and surface for comparison in the measurement perturbations. This type of analysis would be best suited for a numerical study which is beyond the scope of the current paper. Our initial experience with the bone measurements indicates that the transmission configuration is quite stable given that the probes need to be removed at each measurement cycle so that the holes can be drilled deeper into the bone which entails a fair amount of cable bending. In addition, the probes were held in place manually suggesting that the contact with the bone surface incorporates some variability. In spite of these issues, the measurements remain quite stable. These aspects will receive additional consideration during further stages of development.

For the second, errors in the distance directly impact the recovered property values. If we consider a distance for the sample measurement to be on the order of 20 mm and the possible error was ±0.25 mm, this would amount to an error of ±1.25%. From the analysis in Section 2.1, the distance error relates directly to the slope values which would also vary ±1.25%. However, in examining Equation (6), the values for *ε′* go as the square of both slopes (*β* and *α*), such that a 1.25% error in the slopes equates to a 2.5% error in *ε′*. Likewise, because *ε″* goes as the product of *β* and *α*, its error would be on the order of 2.5% when the separation distance changed 1.25%.

Finally, for the actual implementation of this probe technique, it will only be possible to make a microwave measurement at a set separation distance. It will also require a calibration measurement at a set distance while outside of the bone. A fixture will need to be developed that allows for a reasonably close measurement while still keeping the probes within the far field of each other. The calibration technique will need to be robust and convenient to make the entire process feasible. These errors will need to be evaluated once implemented.

## 3. Results

### 3.1. Dielectric Liquid Testing and Analysis

**Figure 2:**
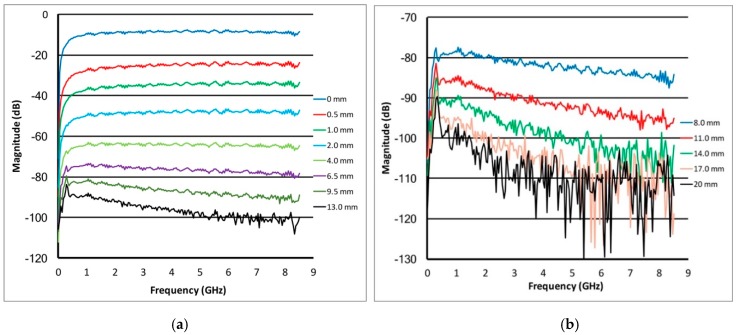
Magnitude plots for the transmission probes as a function of frequency for (**a**) a set of closer spacings (0–13 mm) and (**b**) a set of more distant spacings (8–20 mm), respectively, for an 80:20 glycerin:water coupling bath.

Figure 2a,b show plots of the S_21_ amplitudes as a function of frequency for multiple separations for the 80:20 glycerin:water bath. The graphs are separated into a set of narrowly spaced probes spanning (a) separation distances from 0 mm to 11 mm and (b) for more broadly spaced distances from 8mm to 20mm. Overall the signal strengths are increasingly higher for closer separations. In addition, especially for the lower frequencies and spacings less than 4mm, the signal strength drops off monotonically with decreasing frequency. This is a direct consequence of the opposing open-ended coaxial cables nearly approximating a series capacitor which behaves as a high pass filter. More noticeably, at frequencies below 0.5 GHz, there is a large ripple in the amplitude for separations greater than 4 mm which progressively decrease in size as the separation decreases. This is primarily caused by multi-path signals which can travel along the outer surfaces of the coaxial cables and along the tank:liquid and tank:air interfaces [45]. In these cases, the attenuation due to propagation along these paths can be less than that for signals crossing the gap between the probes to the extent that when they recombine with the desired signals, they can add constructively and/or destructively, consequently, accounting for this rippling behaviour. The phenomenon diminishes at higher frequencies because the attenuation along these alternate paths increases due to the increased liquid conductivity as a function of frequency. This feature also diminishes for closer separations because the desired signal propagating directly across the gap is sufficiently strong to easily overwhelm the unwanted signals. These types of multi-path corruptions are typical of near field experiments and have been documented in Meaney et al. [45,46]. The signals at higher frequencies decrease progressively more rapidly as a function of separation distances than the lower signals. However, within the range of 2 to 6 GHz, the decrease in signal strength as a function of increasing separation is reasonably monotonic with acceptable signal strength even out to roughly 17 mm separation. This is effectively the usable bandwidth for this approach.

**Figure 3:**
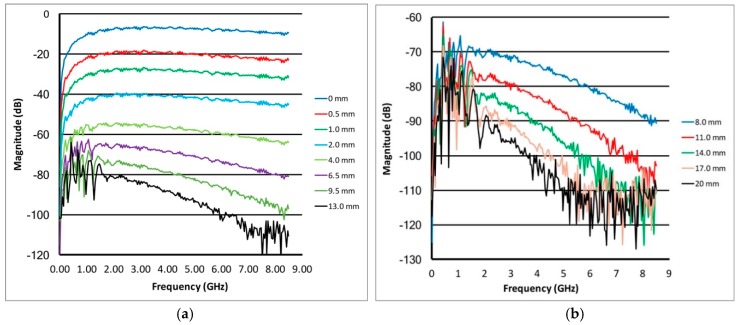
Magnitude plots for the transmission probes as a function of frequency for (**a**) a set of closer spacings (0–13 mm) and (**b**) a set of more distant spacings (8–20 mm), respectively, for a 20:80 glycerin:water coupling bath.

Figure 3a,b shows similar plots of the S_21_ amplitudes as a function of frequency for multiple separations for the 20:80 glycerin:water bath. The graphs are separated into a set of narrowly spaced probes spanning (a) separation distances from 0 mm to 11 mm and (b) for more broadly spaced distances from 8 mm to 20 mm. Overall, the signal attenuation in this liquid is lower due to the lower water content. This is evident where the attenuation for given spacings is less than that for the corresponding levels for the 80:20 glycerin bath cases. In addition, the attenuation roll-off as a function of frequency is substantially more pronounced. Interestingly, the multi-path ripples extend up to roughly 2 GHz for spacings greater than 4mm compared to only 0.5 GHz for the 80:20 case. Even with these unwanted signals, this approach appears to remain viable from roughly 2 to 6 GHz and up to a spacing of 17 mm before becoming compromised by the noise floor.

**Figure 4:**
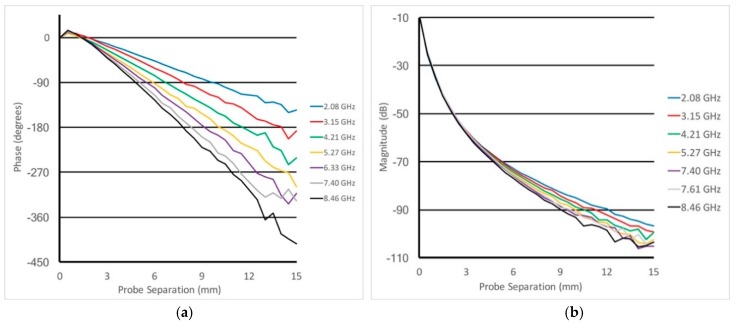

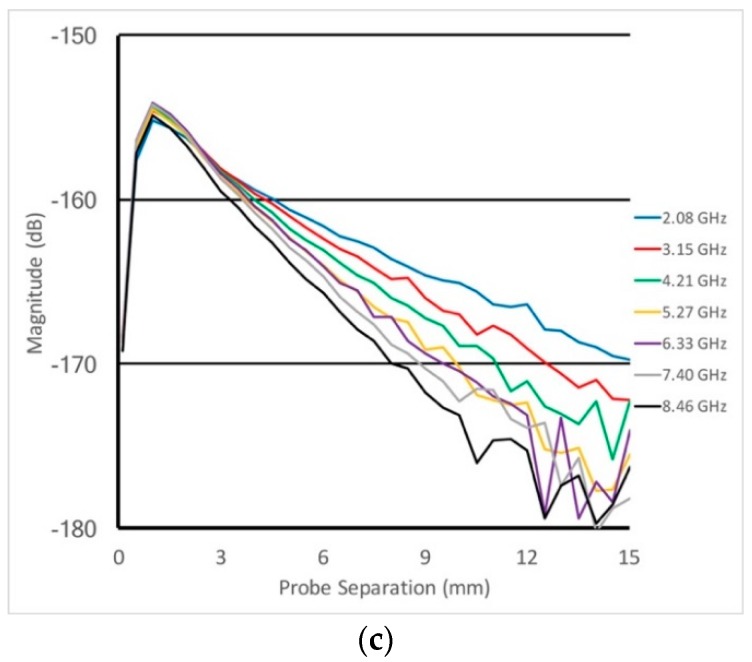
Plots of the (**a**) phases and (**b**) magnitudes as a function of separation distances for several representative frequencies across the 2–8 GHz band. (**c**) shows the magnitude plots after compensation from the technique described in Equation (3).

Figure 4a,b shows the phases and magnitude of the measured signals as a function of separation distance in the 80:20 bath for several of the frequencies within the 2 to 8.5 GHz band. The phases have all been normalized to 0 degrees when the probes are touching. As can be seen, beyond roughly a 1.5 mm separation, the phases are nearly linear with the exception of progressively increasing variability at greater distances associated with the encroaching noise floor. For analysis purposes, the slopes were determined based on least squares fits of the plots to straight lines while eliminating the data points for distances less than 1.5 mm (because of near field effects) and greater than 15 mm (because of noise corruption). For the magnitude cases, the $1/r^{2}$ feature is readily evident. Even with this confounding attribute, it is still clear that the attenuation per unit distance still increases with frequency. Figure 4c shows the same data in Figure 4b but with the $1/r^{2}$ term subtracted out as described in Section II.A. Beyond 1.5 mm and below 15 mm, the resulting curves are roughly linear. The slopes for each were determined utilizing a least squares fit to a straight line.

**Figure 5:**
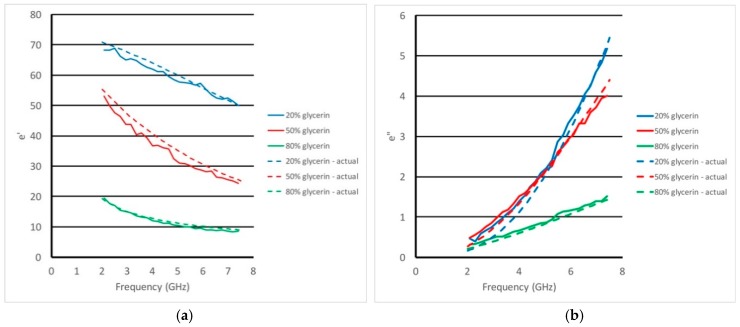
Plots of the recovered (**a**) real and (**b**) imaginary permittivity values using the transmission probe technique compared to the actual property values as a function of frequency for a range of glycerin:water mixtures: 80:20, 50:50 and 20:80, respectively.

Figure 5a,b shows the recovered real and imaginary permittivity values, respectively, over this frequency range for three different glycerin:water mixtures (80:20, 50:50 and 20:80) utilizing the new transmission-based approach compared with ground truth measured using the standard dielectric probe kit from Keysight Technologies. The match is quite good for both properties over the prescribed bandwidth even for the substantial range in properties for these liquids. Interestingly, it also appears that the new technique accurately mimics the characteristic dispersion curvature of the different liquids which can be quite distinct where some are concave upwards and others concave down.

### 3.2. Preliminary Bone Measurements

**Figure 6:**
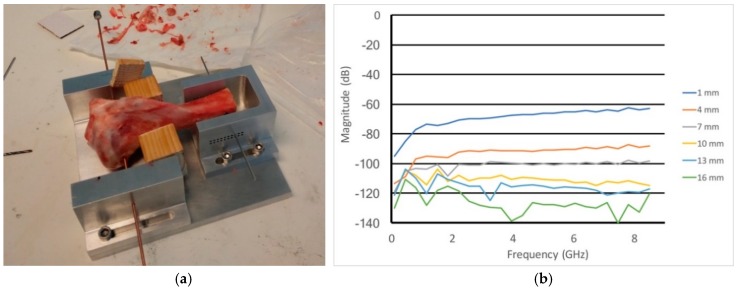
(**a**) Photograph of the bovine bone sample in the measurement test fixture with the coaxial cables extending outward from either side and (**b**) magnitude measurements for the bone sample over a range of separation distances.

Figure 6a shows the test configuration using our transmission probe technique on the trabecular portion of a bovine bone. In this case, the bone was secured with a rod through the cortical portion of the bone. Small diameter holes were drilled into both sides of the bone utilizing guide holes on either side of the fixture. For this experiment, holes were drilled at roughly 1 cm deep from both sides (one goal was to keep portions of the coaxial lines inside the bone to add attenuation to any signal leaking out and minimize multi-path corruption). For each measurement, the amplitude and phase were recorded over our full bandwidth −0.1 to 8.5 GHz in 350 MHz increments. 25 samples were taken for each sweep, the IF bandwidth was set to 10 Hz and the averaging factor was 10. The time for each acquisition was 70 s. After each measurement, the probes were removed and the depth of one hole was drilled 3 mm deeper. The probes were re-inserted and an associated measurement taken. This process was repeated until the probes met in the centre of the bone—note that the drilling side was alternated between each measurement to ensure the final locations were as deep in the bone as possible. The measurements were generally immune to artefacts related to repeated cable motion and bending during the overall process.

Figure 6b shows the amplitude plots as a function of frequency for several probe separation distances (these distances were computed based on the point at which the probes contacted each other and drill depth at each increment). Similar to the graphs for the uniform liquid measurements, the amplitude declines monotonically as a function of separation distance for frequencies greater than 5 GHz. The slopes of the curves as a function of frequency are steeper than that for the 80:20 glycerin:water tests indicating that the attenuation per unit distance is greater. The multi-path corruption appears at considerably higher frequencies than previously observed for the liquid tests. These are likely due to the fact that the propagation modes on the outsides of the coaxial cable do not have sufficient distance within the holes drilled in the bone to sufficiently attenuate. However, there does appear to be a band between roughly 4.5 GHz and 7 GHz where the curves behave well enough for properties to be extracted.

**Figure 7:**
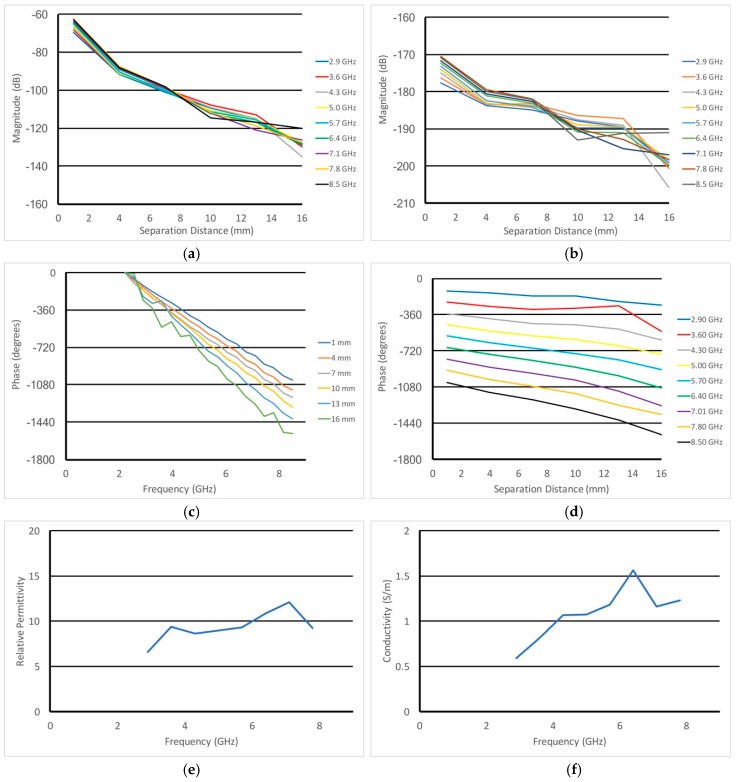
Plots of the (**a**) magnitude as a function of separation distance and (**b**) with the 1/*r^2^* term subtracted out for a set of representative frequencies, respectively. Plots of the (**c**) unwrapped phases as a function of frequency and (**d**) the phases as a function of separation distance. Plots of the recovered (**e**) permittivity and (**f**) conductivity as a function of frequency utilizing the transmission mode technique.

From the data in Figure 6b, Figure 7a,b shows the transposes of the (a) magnitudes and (b) adjusted magnitudes with the 1/*r*^2^ term subtracted out as a function of probe separation distance for representative frequencies. The former plots are relatively well behaved with a slight concave upwards curve to it. The plots in Figure 7b are generally along more of a straight line having had the 1/*r*^2^ factor removed. While there appears to be more ripple in the data, this is somewhat due to a narrower scale than that for Figure 7a. Figure 7c,d show the associated phases for the same measurements. For the plots in Figure 7c, the phases have been normalized to their value for 2.2 GHz and have been unwrapped as a function of frequency (this technique has been explicitly described in Meaney et al. [47]). Figure 7d shows the phases from Figure 7c transposed and plotted as a function of separation distance. With respect to the transmission mode probe algorithm, the slopes of the magnitudes and phases were extracted from the graphs in Figure 7b,d, respectively. The final relative permittivity and conductivity values are plotted in Figure 7e,f as a function of frequency. These values are nominally in line with that reported by Peyman and Gabriel [48] which showed a considerable variation in animal bone dielectric properties as a function of age.

## 4. Discussion and Conclusions

We have demonstrated that utilizing two opposing open-ended coaxial probes, we can accurately recover the transmission medium dielectric properties for quite short distances. This concept is limited in applicability to testing objects where opposing sides can be accessed and the distance is short. However, the technique has important advantages over the classical, reflection-based open-ended coax probe in critical settings. For instance, the reflection based probe’s penetration depth varies as a function of probe diameter and only extends roughly 280 μm for the commercial probes from Keysight Technologies. The transmission-based probes provide a more uniform assessment of the volume circumscribed by the space between the probes. It is also worth noting that these probes operate over a very broad bandwidth, bracketed by multi-path corruption at the low end and the noise floor at the high end.

From an operational perspective, the reflection-based probes are challenging to work with given that they are prone to measurement artefacts due to even slight motion of the connecting cables. This is largely due to the fact that the probes are calibrated to operate at one of the more sensitive locations on the Smith chart—i.e., an open circuit. The contact interface is also critical since even small amounts of intervening air can easily disrupt the desired measurements. It will be crucial to evaluate how sensitive this transmission-based approach is to small air gaps in future studies.

**Figure 8:**
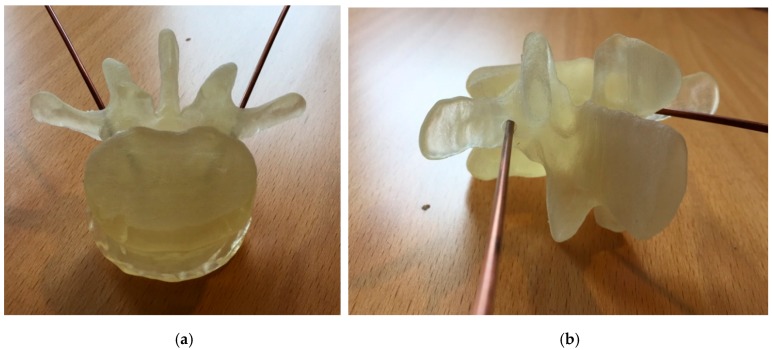
Photographs of a 3D printed human vertebrae (lower lumbar) with two coaxial probes inserted into holes formed through the pedicle arms into the main portion: (**a**) top down view with the probes partially visible with the plastic and (**b**) a side view showing the hole that the probe is inserted into.

The intended application for this technique will be to measure the dielectric properties of vertebrae during surgery to provide an indication of bone health. Figure 8a,b shows two photographs of a 3D printed, lower lumbar vertebrae with two coaxial probes inserted for illustration purposes. As can be seen, the holes through the pedicle arms will be quite small, making it challenging to get anything larger than a small coaxial cable into it. It can also be seen that where the cables terminate inside the bone, their separation distance is only on the order of roughly 1.5 cm. Per our earlier data, it appears we have sufficient signal to detect these measurements. Adjustments will necessarily be made to curve the ends of the open ends so that they face each other. We have extensive experience in these types of alterations. In addition, it will be necessary to mechanically determine the probe separation distances so that the appropriate magnitude and phase slopes can be computed (a reference measurement will need to be made externally to complete the data for the slope calculations). These processes will be designed as part of the product development.

In utilizing the new transmission-based probes in a clinical setting, it will be necessary to record measurements at two different separation distances to calculate the magnitude and phase slopes that are used to derive the dielectric properties. The first measurement will be across the tissue volume of interest. A convenient second option will include the case where the two open-ended coaxial probes directly contact each other outside of the body. One challenge will be to determine the spacing of the probes when positioned across the volume. Various geometrical triangulation techniques will be explored for estimating this distance. A second involves the fact that in the case where the probes contact each other, the fields are in the near field and deviate from their desired linear behaviour. As can be seen from the previous data, the measurement characteristics are reasonably well behaved and it will be relatively straightforward to devise a compensation technique. These issues need to be resolved as part of developing an actual product.

The current results were somewhat limited in total depth because of the noise floor of the measurement vector network analyser (VNA). While most conventional VNA’s have noise floors on the order of −100 dBM, newer models such as the Rohde & Schwarz ZNBT8 system (Munich, Germany) are capable of measuring signals down to −150 dBm. The cost of this system would be prohibitive for clinical applications. However, new technology, including the use of software defined radios (SDR’s) will make these higher dynamic range measurements feasible and at nominal costs [37,49]. In this case, the key will be to be able to add sufficient signal amplification and sampling time to adequately reduce the noise floor. These advances are well within current technology capabilities.

While it is challenging to envision applications for this technique, in collaboration with orthopaedic surgeons, we are applying this approach to testing the bone quality of individual vertebrae during actual bone fusion surgeries. In this context, it is critical that doctors accurately know the strength of the vertebrae before using pedicle screws to fix the spinal column with appropriate instrumentation. Conventional techniques such as dual-energy X-ray absorptiometry (DXA) only provide average values of the systemic bone mineral density which is insufficient with respect to the individual vertebrae for which the strength can vary considerably from bone to bone. The unique aspect of this application is that the surgeons already form a slight hole through both pedicle arms of the structure into the main body of each vertebrae. In this setting, the coaxial probes will be custom designed to fit into the holes such that once deployed, the faces of the probes will be oriented towards each other and usually separated by 2 cm or less. This has the potential to be an ideal application for this approach where it will require regular cable bending and non-ideal probe:target contact. This approach will revolutionize the use of dielectric measurements for in vivo settings.
